# Self-directed learning readiness of Asian students: students perspective on a hybrid problem based learning curriculum

**DOI:** 10.5116/ijme.582e.021b

**Published:** 2016-12-03

**Authors:** Lukas D. Leatemia, Astrid P. Susilo, Henk van Berkel

**Affiliations:** 1 Department of Medical Education, Faculty of Medicine Mulawarman University, Jl. Kerayan, Kampus Gn. Kelua, Samarinda, East Kalimantan, Indonesia; 2 Department of Educational Development and Educational Research, Faculty of Health, Medicine and Life Sciences, Maastricht University, The Netherlands

**Keywords:** Self-directed learning readiness, hybrid problem based learning curriculum, influencing factors

## Abstract

**Objectives:**

To identify the student’s readiness to perform self-directed learning and the underlying factors influencing it on the hybrid problem based learning curriculum.

**Methods:**

A combination of quantitative and qualitative studies was conducted in five medical schools in Indonesia. In the quantitative study, the Self Directed Learning Readiness Scale was distributed to all students in all batches, who had experience with the hybrid problem based curriculum. They were categorized into low- and high -level based on the score of the questionnaire. Three focus group discussions (low-, high-, and mixed level) were conducted in the qualitative study with six to twelve students chosen randomly from each group to find the factors influencing their self-directed learning readiness. Two researchers analysed the qualitative data as a measure of triangulation.

**Results:**

The quantitative study showed only half of the students had a high-level of self-directed learning readiness, and a similar trend also occurred in each batch. The proportion of students with a high level of self-directed learning readiness was lower in the senior students compared to more junior students. The qualitative study showed that problem based learning processes, assessments, learning environment, students’ life styles, students’ perceptions of the topics, and mood, were factors influencing their self-directed learning.

**Conclusion:**

A hybrid problem based curriculum may not fully affect the students’ self-directed learning. The curriculum system, teacher’s experiences, student’s background and cultural factors might contribute to the difficulties for the student’s in conducting self-directed learning.

## Introduction

Self-directed learning (SDL) can be defined as students control their own learning process by using planning, implementation, monitoring, and evaluation. Students identify their learning needs based on the problems they find in the learning process and in the environment, what skills they need to train in, and what information they want to find. They formulate their own learning goals, and then translate them into specific objectives. Students also monitor their own learning processes to ascertain that the plans have been performed correctly and to determine the steps yet to be performed. In evaluation, learners will assess the new skills they have acquired, whether the answers or solutions are satisfactory, as well as the quality of new ideas and knowledge. In this principle, the students are responsible for their own learning experiences.^1^^,^^2^

Many authors agree that the problem-based learning (PBL) can increase the student’s self-directed learning skills. In PBL, students use their prior knowledge by developing a hypothetical-deductive approach to explain the cases. Students realize they can make their learning relevant to their own educational needs by activating their prior knowledge and elaborating on newly acquired knowledge. Students are also equipped with a rich learning environment so that they are able to discover informational resources independently.^3^^-^^5^

However, in Asia, there are still some conflicting perceptions about the effect of PBL on the students’ SDL. On one hand, some schools reported that the benefits of PBL actually increased the students’ SDL skill. Khoo (2003) pointed out that in Malaysia, a majority of students agreed that PBL had encouraged them to seek information and improve their understanding which led to an integration and application of that knowledge. In Sri Lanka, PBL encouraged students to do SDL which resulted in a deeper approach to learning with an increased motivation along with the pleasure of learning.^6^ On the other hand, some authors pointed out the difficulties in conducting SDL in PBL, in particular for Asian students. For example, students were not confident enough to seek information themselves and afraid of confrontation with authority figures. These can lead to a lack of enthusiasm for studies, a lack of motivation or ability to ask questions, and a low participation in class discussion.^6^^,^^7^

In Indonesia, SDL has been an important issue in medical education since the Indonesian Medical Council (IMC) declared the standard and the regulation for teaching and learning processes in 2006. This regulation led all medical schools in Indonesia to change their curriculum from a traditional curriculum, which emphasized teacher-centered learning into a competence based curriculum (CBC) with student-centered, problem-based, integrated, community based, elective, and systematic (SPICES) approaches.^8^ However, many institutions and teachers believed that it was difficult for Indonesian teachers and students to change their behaviors because they had become accustomed to the teacher-oriented methods.^9^^,^^10^ Yet, almost all of medical schools in Indonesia have started to implement a hybrid PBL curriculum even though the evidence of the hybrid PBL curriculum influencing the students’ SDL is still rather inconsistent.^11^

In addition, the implementation of the curriculum was a result of the government enforcement without preliminary reflection on the reasons to integrate PBL into the curriculum. Samarasekera and Karunathilake explained some challenges and problems for students if the schools design their curriculum without careful planning, such as students’ dissatisfaction with PBL methods, and students’ difficulties in moving from a teacher led to a student driven environment in quick sequences.^12^ These factors may contribute to the failure of students to implement a constructive, self-directed, collaborative, and contextual process.^3^

Therefore, evaluation about the appropriateness of the new curriculum in Indonesia, in particular its influences regarding the students’ readiness to do SDL, is still needed. Some questions are addressed in the new curriculum: (1) Does the hybrid PBL curriculum positively affect students’ SDL; and (2) What are the underlying factors that influence students’ SDL? We would assume that students who had more experiences in a hybrid PBL curriculum would have higher levels of SDL readiness. The results of this study can provide important information about the student’s SDL capabilities in Indonesia with some recommendations for improvement.

## Methods

### Study design and participants

This is a combination of quantitative and qualitative studies which were conducted at five medical schools in Indonesia: Faculty of Medicine Riau University (FMRU), Faculty of Medicine Pembangunan Nasional University (FMPNU), Faculty of Medicine Mulawarman University (FMMU), Faculty of Medicine Samratulangi University (FMSU), and Faculty of Medicine Udayana University (FMUU).  We chose these particular universities because they were similar in age and curriculum structures.  They were also representative of the five major islands in Indonesia: Sumatera, Java, Kalimantan, Sulawesi, and Bali. These five universities used a traditional curriculum formerly, then changed to a hybrid PBL curriculum in 2006 and 2007.

PBL is used as one method besides the other conventional learning methods such as classical lectures, laboratory sessions, and skills training. PBL activities represent 40% to 50% of learning activities. Samarasekera and Karunathilake named this format a hybrid PBL because PBL is combined with other different teaching-learning methods.^12^ The number of members in each PBL session is 8 to 10 students. The scenarios of the tutorial are paper based and given before the module begins. The scenarios and the schedule of the module are available in the students’ guidance book. The schedule also includes a protected time for self-study.

Every week students have one session of PBL, which consists of two small group meetings and a plenary discussion in a large class at the end of the week. In between two small group meetings, students have three days to conduct self-study to expand on the learning objectives they decided on in the first meeting. In the plenary discussion, two groups will present their tutorial discussion results and then all students share what they have learnt in their own groups and ask questions about anything they do not understand. In this meeting, students discuss all questions from other students and share some new information that other groups have not discussed previously. The content experts attend this plenary discussion and clarify the problems that arise at that time.

The participants in the quantitative study were all students who had experienced the new curriculum (students of year 2007 to 2010) and provided a written consent before filling in the questionnaires. The questionnaire was used to determine the SDL readiness level (low or high).

In the qualitative study, we conducted FGDs with three groups consisting of 6 to 12 students. Groups were based on their level of SDL readiness (high, low and mixed group). Participants were chosen randomly from each category.^13^ Considering the geographical and logistic reasons, we conducted the qualitative study only with students from FMMU.

The ethical clearance was granted by the FMMU Board and Ethical Committee. No physical risk was found in this study. All of the students were given written and oral information about this study before being asked for their consent. The students were free to decide in joining and resigning this study at any time with no consequence to their learning assessments and activities. All student identities were kept confidential and not mentioned in the transcripts, study reports, or publications related to this study.

### Data collection

In the quantitative study, the data was obtained from participants by directly distributing and collecting the questionnaires about their levels of SDL readiness. The instrument used in the quantitative study was a questionnaire consisting of 40 items about the students’ SDL adopted from Self Directed Learning Readiness Scale (SDLRS). SDLRS is used to measure the degree of the individual’s attitudes, abilities and personality needed for SDL. SDLRS is divided into three categories: self-management, desire for learning, and self-control.^14^

Validation and reliability analysis for the questionnaires had been done before the students’ levels of SDL readiness were decided. Validation was conducted by translation from the original language (English) into Indonesian and back translation into English.  Those processes were conducted by an English teacher and some medical teachers who have postgraduate degrees in medical education.

Reliability analysis was conducted for the three components: (1) self-management, (2) desire to learn, and (3) self-control. The analysis showed strong relations among the items in each component. The reliability analysis showed that the levels of internal consistency of all items are acceptable; with the value of Chronbach’s coefficient alpha for total score and component 1, component 2, and component 3 were more than 0.70 (0.905, 0.745, 0.825, and 0.836 respectively). Table 1 shows the measure of central tendency and the Chronbach’s alpha of each component and all items. A total score greater than 151 indicates readiness for SDL.

These scores closely resemble ones in the original paper. In the original analysis, a total score of more than 150 indicated readiness for SDL; and Chronbach’s coefficient alpha of the total score was 0.924.^14^ 

In the qualitative study, we used FGDs to explore the factors influencing their level of SDL readiness. The students in each group shared freely their experience in SDL. Two researchers acted as the moderator and the note-taker in the FGDs. The FGD processes were recorded using both audio and video recorders. Before the discussion started, the moderator gave a brief introduction, the objectives of the study, and the rules of the FGD. All of these statements as well as the trigger questions of the discussions were documented in the discussion guidance.^13^

**Table 1:** Total score and measures of central tendency, and reliability analysis of the questionnaires

Central tendency measures		Components		Total
	Self-management	Desire for learning	Self-control	
Mean	45.43	47.80	57.8	150.95
SD	5.06	5.20	6.56	14.36
Median	46	48	57	151
Mode	46	46	57	150
Minimum	30	19	31	91
Maximum	58	58	73	188
Chronbach’s alpha	0.745	0.825	0.836	0.905

### Data analysis

The SDL data from the questionnaire was grouped into low and high levels and then analyzed by using univariate analysis. The table distribution and the proportion of students in each variable were analyzed using SPSS 15.0 program and Microsoft Office Excel. The FGD results were recorded and transcribed and analyzed by creating codes and a coding tree, and then distributed into the tables.

The transcripts were analyzed by two researchers as a measure of triangulation.  They started with an open coding process independently and discussed the analysis to define a coding tree. Afterward the coding tree was used to recode the transcripts.  Differences were discussed until a consensus was reached. The emerging themes were structured in tables.

## Results

From 1,178 students involved in this study, we found 562 (42.3%) students have a low-level of SDLR and 614 (60.5%) students have a high-level of SDLR.

**Table 2:** SDLR level of students in 5 medical schools in Indonesia (N=1178)

Variable		SDLR Level			
		Low (n=562)		High (n=614)	
		n	%	n	%
Universities					
	FMMU	120	53.3	105	46.7
	FMRU	140	55.3	113	44.7
	FMSU	48	40.7	70	59.3
	FMUU	46	37.1	78	62.9
	FMPNU	208	45.6	248	54.4
Year of students (Batches)					
	First Year (2010)	168	43.1	222	56.9
	Second Year (2009)	148	46.0	174	54.0
	Third Year (2008)	166	53.7	143	46.3
	Fourth Year (2007)	80	51.6	75	48.4

FMPNU, a university in Jakarta, had the most respondents while FMUU and FMSU, Universities in Bali and North Sulawesi respectively, had less respondents. The number of students from year 2009 who had a higher-level of SDLR was more than those of the students in year 2007 and 2008 but the number was less than those of the students in 2010 (Table 2). 

From the FGD analysis, we found that the external and internal factors were influencing the student’s SDL. The external factor is the instructional process that often acts as a facilitator while the internal factor involves personality aspects that refer to the learner’s desire or preference in assuming responsibility for learning.^1^ The external factors that influenced the student’s SDL are the PBL process (such as the tutors, fellow students in the group, and the PBL methods), the assessments and the learning environments (such as family members, dorm mates, learning facilities, and learning atmosphere). The internal factors were perception to topics, lifestyle, habits, and mood.

In this study, most of the students felt that the PBL method forced them to do independent learning by seeking and reading many references by themselves and evaluating their own learning.

“The PBL system greatly spurred my passion for learning more than if I had just taken the classes. With the PBL system, I understand it better and that makes explaining it easier.” (Student 1, group 2)

Some students agreed that they were more active if there was an increase in members studying and actively involved in the tutorial. Motivations were increased when their fellow students studied more than they had and shared their many references. Likewise, the motivation decreased when many members were not actively involved in the tutorial and there was no feedback from the tutor to the students.

“During the tutorial my friend explained many things and it motivated me to study. I had to catch up with self-learning so that I would not be left behind.” (Student 3, group 2)

“In the tutorial, I could compare my learning ability to my friends. This triggered self-learning as well as helping to evaluate the results of my study.” (Student 1, group 3)

In their perception regarding the tutors, students felt that some tutors interrupted their discussion and added instruction that limited their discussion topics. Or some tutors only attended the tutorial without clear instruction or were too passive during the tutorial process.

“I think PBL can indeed be used to help the students learn things that are difficult, but sometimes the concept of PBL is not stimulating enough. For almost 2.5 years I was forced to study using PBL, I was bored because the same method was used each week. In addition, students are not encouraged by the tutor to understand something. The tutor sometimes said, “do not learn too much” or “this is not needed to be learned”, when we actually wanted to learn it. Because our discussion was assessed by tutor, ultimately we had to follow what the tutor wanted”. (Student 2, group 2)

In the PBL process, a tutor facilitates the discussion process while assessing the student’s participation and ability to share their opinion. This motivated and forced students to be involved in the discussion and to search for and share many references.

“PBL in the design here is "forcing" students to learn. That is, if a student did not speak in the discussion he/she would receive a low score, but if the student was involved in the discussions then he would receive a higher score.” (Student 2, group 3)

In this study, some expressed a lack of confidence with their self-study results. Thus they needed good responses and feedback from the tutors rather than the tutor interrupting during the discussion process. In their opinion, it was good if the tutors were given explanations about the learning process of the tutorial.

Some assessments in learning methods, such as pretest and questions asked by teachers while teaching, also increased the student’s motivation to study.

“Pretests in the skill lab and laboratories spurred me to study at home to prepare for the pretest itself and learn about the topics to be practiced. While in the lectures, if the teachers often asked as to whether the students understood or not, then it would motivate me to learn in advance and anticipate the questions from the lecturer.” (Student 4, group 2)

However, this assessment became negative when some students felt that it was subjective. Tutors did not give a distinct score and feedback between passive and active students. The students assumed it was useless to be involved actively in the discussion and this decreased their motivation to participate (the step 6th of the 7 jumps). They still showed that their learning orientation was in the assessment results. 

“Sometimes the tutors gave students subjective judgments. The tutor already had a good perception of the student from the beginning and continued to carry this over throughout the PBL processes even though the student was not active in the discussion at the time. The student still received a good score. I do not think this is fair and it decreases my interest in learning”. (Student 1, group 1)

A non-constructive learning environment is another external factor that students shared. Noisy environments from family members in their parent’s house or from their dorm mates and surrounding dormitories, decreased their motivation to study. They had difficulties studying in those environments:

“My family members are not used to studying. When I was just starting to learn, they always disturbed me. For example, they would ask me to go for a walk or mocked everything I said. This often decreased my motivation to study”. (Student 2, group 1)

Studying in FMUM’s library also had some problems because many reference books were not available.

“We have Internet connection or Wi-Fi but the reference books in the library are lacking”. (Student 3, group 1)

However, support from their parents and many questions from their dorm mates about their health conditions also motivated them to learn more:    

“Friends in our dormitory often asked me about the problems associated with their health. This increased my motivation to study. If I knew the answer I would tell them, but if I did not know I would have to find the answer from text books or internet”. (Student 5, group 2)

“I think that parental support was very helpful in increasing my motivation to learn. My parents are the types who are open minded and always ask about my learning needs. Because of their kindness, I was encouraged to learn more. I felt guilty if I played more because my parents were very kind to me”.  (Student 6, group 2)

The internal factors influencing student’s SDL are lifestyle or habits, perception to the topics, and mood. In some cases, the internal and external factors are related to each other.

Student’s lifestyles or habits, such as their daily time management and SDL experiences in their senior high school, also influenced them and motivated them to participate in the SDL. Some students agreed that they used to do SDL in their senior high schools.

“I was already used to reading lots of books before studying in this medical school. In high school I used to read many books independently to prepare our group discussion every week. My teachers in high school often did not provide materials in depth, so the students had to study more on the topics. If I felt that I needed these topics, I would definitely look for as many references as possible to learn and read without being affected by the learning methods.” (Student 7, group 2)

Nevertheless, some other students had difficulties studying in the PBL process.  They were required to arrange their own study because in their senior high schools, their teachers often arranged and scheduled their studies for them.  They were accustomed to studying a few days before the final assessments began. The questions were taken from the dictates and hand outs and they could answer the questions by just reading the papers. This decreased their motivation to seek other reference books and learn from many other sources.    

Another factor is perception to the topics. It means they would be eager to study if they knew that the topics were important to their future and applicable in their daily lives. This would increase their motivation to learn these particular topics before others that they were not interested in:         

“I am more motivated to learn if I know what the ultimate goals of the topics are and what its implementation is in my daily life. For example, topics that relate to medical skills. I can implement many skills in my daily life, such as helping people who are involved in an accident”. (Student 4, group 1)

“I prefer studying the topics that I like, no matter what is the method of learning, e.g. lectures, laboratories, tutorials, or skills laboratory. If I like the topics, it motivates me to learn more”. (Student 3, group 3)

Some students admitted that they would study if they were in a good mood at the time. In other words, they would learn depending on their mood:

“I learn some topics depending on my mood. When I am in a good mood, I can study highly motivated. But If I am in a bad mood, I cannot be forced to study. I cannot understand the matter even though I have already read about it many times. My study time is brief when I am in a bad mood”. (Student 4, group 3)

## Discussion

The main concern from some of our teachers was the inability of our students to perform SDL. Their assumption was that most students could not implement the PBL method because they were not ready to do independent learning. This study was conducted to give evidence about the level of SDL readiness of students who had experiences in PBL and the factors influencing it and then to give some recommendations based on the analysis. The levels of the student’s SDL were identified based on the students’ readiness to conduct SDL. It was predicted that students who had more experiences in PBL would have a higher level of SDL because like many authors stated, PBL cooperatively and positively affects the students’ SDL.^3^^,^^11^

However, the results of this study showed that only half of the respondents had high-level SDL readiness, a similar trend also occurred in each batch. There were still many students not ready despite experiencing many PBL sessions. This study also showed that the percentage of high-level SDL tends to be lower in the senior students compared with more junior students. This raises a presumption that the experience may not affect the level of SDL.

Maung, Abas and Abdullah also found this phenomenon in their study and pointed out that students who just entered the university usually have a greater anticipation and expectation toward a new learning environment.^15^ The orientation activities, where the new students are taught and trained PBL process might support students having greater anticipation and expectation toward a new learning environment and begin to adopt the new learning method. However, after adaptation to the learning environment, the students faced a “honeymoon effect” meaning the self-directed behavior will fade away after a period of time. After passing this phase, students would enter an acceptance phase in which they would reflect their learning process and get a deeper appreciation for SDL as a useful learning method.^15^^,^^16^

We argue that there are some reasons why the students find it difficult to increase their SDL. The curriculum system and teacher’s experiences still emphasize a teacher-centered approach. This system also still focuses on summative assessment and memorizing facts. Student’s background and cultural factors also contribute to a decrease in the student’s motivation to conduct SDL.

One of the factors is that the methods of learning still tended to be a teacher-centered approach. Although the curriculum has been applied based on student-centered approach, teaching in large classes, laboratory classes, and skills laboratory classes in the traditional ways are still conducted. With many traditional learning methods students usually have limited opportunities to pursue SDL.^7^^,^^17^ The problems and the challenges occurred when the hybrid PBL curriculum was designed without much consideration to the overall schemes and only focused on how the PBL tutorials should be slotted in the curriculum.^12^ This might lead to conflict of intention and philosophy. Students do not realize the value of SDL in their learning process and only use it when required, such as in the PBL process.^12^^,^^18^

How to conduct PBL properly is also important to stimulate SDL with students. Dysfunction in all aspects of the PBL, such as the problems with the stimulus of learning, tutors as facilitators, group work as stimulus for interaction, assessments as part of the examination system, information resources, and evaluation employed will lead to decrease of student commitment, increase cynicism, and student absenteeism.^3^^,^^5^^,^^18^^,^^19^  

Another factor that inhibits SDL is the teacher’s own learning experience which was still teacher-centered. It is easy for the teachers to revert to the system they have experienced before. When the system of teacher-centered is changed, teachers should adapt to a different role where students are assisted to learn and find their own learning style, rather than act as a main source of information for the students.^10^^,^^18^ That is the reason why students in this study felt that the tutors often intervened in the process of discussion and restricted discussion topics. This would decrease the student’s motivation to be actively involved in the discussion and to study the topics more in depth. The interventions made by the lecturers would lead to a lack of ability for students to find references by themselves and likely be unable to express their opinions confidently and tend to wait for the achieved learning objectives. In addition, the teachers who had experience in teacher-centered learning tend to foster traditional methods in the curriculum. Meanwhile, student centered innovations in the big classes and laboratory classes were hardly conducted.^10^

Assessments conducted for the students were considered not supportive for SDL. Summative assessments carried out in the tutorial was still considered a teacher-directed approach and perceived as a threat rather than a trigger for their study. Students were not intrinsically motivated. They would present but there was not actual participation.^18^ Formative assessment also was not optimally implemented. Not all tutors can provide good feedback and giving incorrect feedback will lower the student’s motivation to study.^18^^,^^20^ Examinations are also not able to improve the students’ SDL if many test items were on a recall level and sourced from handouts and dictates. This caused students often to simply rely on their study from handouts and dictates as the main sources of reference. The questions, which contain memorized factual information will fail to lead students to study deeper, to learn deep conceptual understanding, to solve meaningful problems, and the like.^21^ This might also contribute to student learning orientation, which becomes simply to pass the exam and get a high score.^22^    

Student background and cultural factors might also influence students motivation to achieve SDL behavior.^19^ In Indonesia, paternalistic culture that parents or other family members influence the students’ decision to choose a course or studies is also another inhibiting factor. For example, because a medical doctor is seen as a prestigious profession in our community, parents who are medical doctors often want their children to be a doctor too although the real interest of their children may be something else. This often leads students to a lack of interest in studying in medical school. The other cultural issue is the perception that teachers are powerful figures who know everything and should be listened to, was adopted by students from their earliest school experiences. In the Indonesian culture, a teacher is called “guru”, that mean a reliable person who has to be followed or obeyed. Students are viewed as persons who know nothing and do not need to argue, to think independently and critically, to find information from books or other resources, or even to find other learning strategies that are more suitable to their learning styles. This perception is passed down from generation to generation.^9^

From this study, we recommend that the design of the hybrid PBL curriculum should be improved. The faculty should identify the important transition themes, develop appropriate cases and align the PBL activities with other teaching activities. Dolmans, et al., recommended to be consistent with student directed education. All components in the educational program, such as the teachers who are involved in the conventional methods, tutors, the problem used in the tutorial, and the evaluation employed, should be consistent with a constructivist view on human learning. Although lectures and laboratory classes are still conducted, teachers can create their learning activities in a constructivist view. The teachers should help learners to identify their beliefs and work with them to solve their problems in understanding.^18^

The difficulties also are different between students who have been accustomed to student-centered methods and those with teacher-centered methods. In other words, problems of students who are experts in self-regulated learning are different from problems of novice students. It needs careful consideration to be transformed from externally-guided students to a more student self-guidance.^2^^,^^3^ This can be achieved by facilitating student-student and student-teacher interaction by using reflective feedback to enhance the atmosphere of discussions, providing critical feedback related to the learner’s contributions, and challenging the learner’s sometimes naïve conceptions. The teacher becomes a facilitator of learning rather than a giver of information.^23^

Therefore tutors’ skills have to be improved. Tutors without enough experiences will lead to less commitment and more disruptive behavior.^19^ Tutor training is recommended to increase tutor knowledge about PBL and human learning and skills in tutorial. Musal, Abacioglu, Dicle, Akalin, Sarioglu and Esen pointed out that tutor training conducted in Dokuz Eylul School of Medicine (DESM) Turkey for four days could increase the knowledge and skills of the tutors. Thus, such training was a prerequisite for those before working as tutors.^24^

There are some limitations that we found in this study. The main limitation is that FGDs were only conducted in FMMU because of geographical and time constraints.  Secondly, this study described only the student’s SDL levels at one time without following up on their progressions every year. A cohort study to ascertain the progress of students in having SDL behavior and another study on students who have different learning approaches are needed in the next studies in order to get a model of learning methods, which are suitable for the schools. Some components of PBL, such as the tutor, the scenarios, and the member groups can be evaluated further to uncover problems and decide on solutions in implementing PBL.  

## Conclusions

A hybrid PBL in the new curriculum produced only half of the number of students conducting SDL in the learning processes. The curriculum system and teacher’s experiences still emphasize a teacher-centered approach. The assessments delivered still focus on summative assessment and memorizing facts. Student’s background and cultural factors still contribute to a decrease in the student’s motivation to conduct SDL. The consistency with constructivism on human learning by creating student centered approaches in the conventional learning methods, PBL evaluation and improvements, particularly increasing knowledge and skills of the tutors by conducting tutor training and assessment improvements, are recommended. Teachers can perhaps be advised to strengthen their abilities as facilitators rather than simply information providers.

### Acknowledgements

We would like to thank the Dean and the ethical clearance committee of Mulawarman University who have given us the opportunity to conduct this study and the students who participated. Also thank you to Eni Karmila from FMRU, Nur Fitri from FMPNU, I Wayan Sumardika from FMUU, and Herlina from FMSU, who contributed to this study to collect the quantitative data at their university.

### Conflict of Interest

The authors declare that they have no conflict of interest.
